# Alzheimer’s disease and depression in the elderly: A trajectory linking gut microbiota and serotonin signaling

**DOI:** 10.3389/fpsyt.2022.1010169

**Published:** 2022-11-30

**Authors:** Debora Cutuli, Giacomo Giacovazzo, Davide Decandia, Roberto Coccurello

**Affiliations:** ^1^Department of Psychology, University of Rome La Sapienza, Rome, Italy; ^2^European Center for Brain Research, Santa Lucia Foundation IRCCS, Rome, Italy; ^3^Institute for Complex Systems (ISC), National Council of Research (CNR), Rome, Italy

**Keywords:** dementia, Alzheimer’s disease, aging, neuropsychiatric symptoms, depression, gut microbiota, tryptophan, serotonin

## Abstract

The occurrence of neuropsychiatric symptoms in the elderly is viewed as an early sign of subsequent cognitive deterioration and conversion from mild cognitive impairment to Alzheimer’s disease. The prognosis in terms of both the severity and progression of clinical dementia is generally aggravated by the comorbidity of neuropsychiatric symptoms and decline in cognitive function. Undeniably, aging and in particular unhealthy aging, is a silent “engine of neuropathology” over which multiple changes take place, including drastic alterations of the gut microbial ecosystem. This narrative review evaluates the role of gut microbiota changes as a possible unifying concept through which the comorbidity of neuropsychiatric symptoms and Alzheimer’s disease can be considered. However, since the heterogeneity of neuropsychiatric symptoms, it is improbable to describe the same type of alterations in the bacteria population observed in patients with Alzheimer’s disease, as well as it is improbable that the variety of drugs used to treat neuropsychiatric symptoms might produce changes in gut bacterial diversity similar to that observed in the pathophysiology of Alzheimer’s disease. Depression seems to be another very intriguing exception, as it is one of the most frequent neuropsychiatric symptoms in dementia and a mood disorder frequently associated with brain aging. Antidepressants (i.e., serotonin reuptake inhibitors) or tryptophan dietary supplementation have been shown to reduce Amyloid β-loading, reinstate microbial diversity and reduce the abundance of bacterial taxa dominant in depression and Alzheimer’s disease. This review briefly examines this trajectory by discussing the dysfunction of gut microbiota composition, selected bacterial taxa, and alteration of tryptophan and serotonin metabolism/neurotransmission as overlapping in-common mechanisms involved with depression, Alzheimer’s disease, and unhealthy aging.

## Introduction

In aging populations and due to the increasingly prolonged lifespan of people, “brain aging” has become an increasingly important research subject in recent years. Many studies have explored the expanding gap between chronological age and the estimation of brain age, which is attributed to the acceleration of brain aging processes (1). Brain aging and neurodegeneration are leading constructs for the study of dementia, mild cognitive impairment (MCI), and neurological disorders such as Alzheimer’s disease (AD) in the elderly (2, 3). Brain aging is positively associated with late-life depression (LLD) and anxiety disorders in the elderly, for instance, due to the association between depression, oxidative stress, and neuroinflammation (4–6). Nevertheless, brain aging is more pronounced and accelerated in major depressive disorder (MDD), appearing to be a precipitating factor in defective brain morphology, neuronal senescence, and microglial disease (7–9).

This review focuses on the emergence of neuropsychiatric disorders (NPDs) (e.g., depression, apathy, anxiety, irritability, delusions) in patients with declining brain functions from MCI to early dementia and progression to AD (10, 11). Because of the key importance of affective and neuropsychiatric symptoms (A-NPSs) as early predictors of the progression from MCI to AD (10, 12, 13), the present narrative review will account for the comorbidity between NPDs and cognitive deterioration in older people, taking a different perspective by examining gut microbiota (GM) as a driving force contributing to aging and comorbidity between A-NPS and cognitive decline.

Inflammaging, which includes systemic inflammation, immunosenescence, and neuroinflammation, is a “silent killer” of healthy aging. The GM composition in elderly people undergoes pathophysiological changes in terms of reduced diversity of commensal micro-organisms and decreased abundance of beneficial bacterial phyla (14–16). Hence, gut dysbiosis, intestinal inflammation, and microbiota unbalance in aging are interconnected points that connect inflammaging and intestinal immune response to neuroinflammation and alterations of microglial homeostasis as potential underlying factors in A-NPS and dementia comorbidity.

## The gut microbial ecosystem across aging, dementia, and neuropsychiatric symptoms

### Crosstalk between intestinal microbial homeostasis and host immunity

The human GM is considered to be a “superorganism” because of the impressive number (10^14^) of commensal micro-organisms it contains (17). It is composed principally of four main bacterial phyla (i.e., *Bacteroidetes*, *Firmicutes*, *Proteobacteria*, and *Actinobacteria*) but also by *Fusobacteria*, *Verrucomicrobia*, and *Cyanobacteria* phyla as the remaining bacteria population (18) as well as by *Archaea*, yeasts, fungi, protozoa, metazoans, and viruses (19). The GM exerts multiple functions such as food digestion, absorption, and nutrient extraction by also generating, after bacterial fermentation, bioactive metabolites in the form of short chain fatty acids (SCFAs), and supervising the energy metabolism of the host (20, 21). The GM contributes to immune system development and regulation of the host’s immune homeostasis by modulating the functioning of both innate and adaptive immunity (22, 23). Alterations of GM stability and diversity, and an imbalance of composition can perturb the microbial ecosystem (i.e., dysbiosis) and may severely affect the immune system response and host immunity. These alterations can generate gut inflammation and disrupt the function of the intestinal epithelial barrier, which means higher permeability and loss of mucosal barrier integrity. Although damage to the intestinal barrier can be triggered by diet-derived metabolites, pathogens, and food antigens (24, 25), dysbiosis is also implicated in the dysfunction of mucosal barrier components and integrity (26). Either in the case of the effects of dietary factors on intestinal permeability (24, 25) or in the case of diet-induced dysbiosis (27, 28), the final result is always an elevated risk of systemic inflammation and metabolic endotoxemia with the associated release of antigens, such as lipopolysaccharide (LPS), both in animal models (27, 28) and in patients (29).

In bidirectional gut-brain communication, there is bidirectional crosstalk and reciprocal influence between the immune system and intestinal microbial homeostasis that affects brain physiology. Such an interplay requires the intervention of *gut sensors* such as pattern recognition receptors (PRRs) (e.g., Toll-like receptors, TLRs), which detect foreign micro-organisms and pathogens by the intestinal epithelial cells (IECs) and by the cells of the innate immune system (30). The production of specific metabolites (such as SCFAs) by commensal bacteria enables the communication between GM and intestinal macrophages (31). Other examples of innate immunity-GM crosstalk come from the role of TLRs and their expression in the intestinal epithelium. The non-specific response to GM antigens and pathogens-associated molecular pattern (PAMP) mediated by PRRs is also paralleled by the crosstalk between antigen-specific adaptive immune responses and GM (32). A case of crosstalk is given by the immunomodulatory polysaccharide A (PSA) produced by the *Bacteroides fragilis* (*B. fragilis*), which can be recognized by TLRs and shape the host immune system (33), also interacting with TLR2 on CD4^+^ T lymphocytes to stimulate T regulatory (Tregs) cells to secrete the anti-inflammatory IL-10 cytokine and prevent gut inflammation and ulcerative colitis (34, 35). Similarly, PSA administration was also shown to confer neuroprotection against encephalomyelitis in a murine model of multiple sclerosis (36). Interestingly, neuroprotection was suppressed in IL-10-deficient mice (36). PSA-treated mice also develop resilience against experimentally induced viral encephalitis and brainstem inflammation *via* IL-10 secretion by CD4^+^ and CD8^+^ Tregs cells (37).

This excursion into the crosstalk between microbial communities, mucosal immune function, and immune response helps us to convey the impact of dysbiosis on host immune homeostasis and accounts for the development of both autoimmune and neurodegenerative diseases, including AD (38). This link is brightly demonstrated by the alleviation of amyloid-β (Aβ) neuropathology in germ-free AD-like APPPS1 mice (39) as well as by the possibility of converting germ-free mice in AD-like mice by transplanting fecal samples from an AD patient (40).

### Connecting gut microbial dysbiosis to Alzheimer’s disease pathogenesis

The role of GM in AD is of particular relevance in accounting for Aβ accumulation in the brain of patients diagnosed with sporadic AD. Indeed, some interesting associations have been established between selected bacterial species in the GM of AD patients (e.g., *Escherichia*/*Shigella*), amyloidosis, and expression of pro-inflammatory cytokines in the blood such as IL-6, IL-1β, CXCL2, and NLRP3 inflammasome (41). In AD-like APPPS1 mice a positive correlation between *Pseudomonas*, *Odoribacter*, *Anaerofustis*, and brain Aβ deposition was found (39). AD patients and murine models showed alterations in the gut microbial taxa (39–42). Different studies reported the abnormal increase of Gram-negative *Bacteroidetes* described in AD patients, which is linked to the production of pro-inflammatory transcription factors and neurotoxins that have a deleterious impact on blood-brain barrier (BBB) permeability, Aβ accumulation and chronic inflammatory signaling (42, 43).

The fact that GM dysbiosis can contribute to AD pathogenesis strengthens the idea that sporadic late onset AD (LOAD) may have an infectious etiology is the case for infection driven by neurotropic viruses such as Human herpesvirus 1, bacteria such as *Chlamydia pneumoniae*, or fungi such as *Candida albicans* (44–46). However, it is also known that LPS, the major component of a Gram-negative bacteria cell wall, can interact with the TLRs (such as TLR2 and TLR4) expressed on both microglial cells and the intestinal epithelium (47), providing a link between the immune system, PRRs, and the ability to recognize micro-organism-associated molecular patterns. Dysbiosis is responsible for the damage of the mucosal barrier and systemic inflammation, metabolic endotoxemia, and LPS release. Elevated levels of *B. fragilis*-derived LPS have been found in the hippocampus and neocortex of AD patients (42) as well as overexpression of microglial TLR2, TLR4, and LPS receptor CD14 in both AD patients and AD-like mice models (48, 49). Moreover, neurotoxicity induced by Aβ accumulation is linked to persistent microglia activation that is dependent on the interaction with CD14, TLR2, and TLR4 (50). Aβ interaction with the TLR4/CD14 complex on brain microglia has been suggested as a key mechanism in AD pathogenesis (51).

Another interesting fact is the similar pattern of expression of inflammatory genes triggered by Aβ peptide monomers such as the Aβ_42_ toxic fibrils or by LPS endotoxin-induced immune response (52). To recognize the link between intestinal bacteria, immune system activation, and AD pathogenesis it is important to understand that amyloid fibrils can be preserved as PAMPs within the biofilms produced by different bacterial phyla such as *Bacteroidetes* and *Proteobacteria* (53). For instance, the extracellular matrix of the biofilm of *Escherichia coli* (*E. coli*) is largely made for the amyloid curli, which may form amyloid fibrils (54). However, amyloid fibrils can also be generated from other GM members and amyloid-producing bacteria, including *Streptococcus*, *Pseudomonas, Salmonella typhimurium, Mycobacteria*, *Klebsiella*, and *Bacillus* species (55, 56). Because of dysbiosis and increased permeability of the intestinal barrier, microbial signaling and bacterial amyloidosis can trigger inflammation through the indirect production of pro-inflammatory cytokines and chemokines by immune cells distributed in the lamina propria of the submucosal gut compartment (57). Pro-inflammatory cytokines can access the brain *via* circulation transport across the BBB (58), which is exacerbated in the case of inflammatory agents or immune-active molecules that contribute to the disruption of BBB tight junctions (59). Moreover, systemic inflammation and peripheral pro-inflammatory cytokine production are processes paralleled by the cytokine production from microglial cells, as demonstrated by the activation of TLR4/CD14 receptors and the resultant increase of NF-κB (50). GM-produced cytokines may reach the brain *via* the enteric nervous system and the vagus nerve communication pathway (60). In addition, gut bacteria biofilm-derived amyloid fibrils can promote Aβ deposition by cross-seeding with other amyloidogenic proteins. Indeed, *E. coli*-derived amyloid protein curli was shown to be able to cross-seed with amyloid fibril protein A and aggravate amyloidosis in mice (61).

### Dualism between intestinal microbial ecosystem and serotonergic metabolism in the elderly: A “gut- centric” perspective of Alzheimer’s disease

Age progression is a crucial factor determining the composition of the hosted microbiome (62).

The ability to maintain a “healthy microbiota” may determine healthy aging (63). One of the key aspects of the GM transformation across the lifespan is associated with changes occurring during the first 3 years. During this time all the phases of early neurodevelopment can be influenced by GM composition (64, 65), which, together with dietary factors, exogenous stressful events, and exercise, can determine the future pathogenesis of NPDs such as autism spectrum disorder (66, 67). This period is followed by a prolonged phase of stabilization of the GM ecosystem (68). GM diversity decreases with aging, along with a reduction of beneficial species such as *Bifidobacteria* and the parallel-growing colonies of so-called opportunistic species (e.g., Desulfovibrionaceae) and pathobionts (e.g., Gram-negative Enterobacteriaceae bacteria) contributing to inflammaging (69, 70). Healthy aging is associated with a progressive, but not severe, loss of commensals (e.g., *Faecalibacterium*, *Prevotella*, and *Bifidobacterium*), which are often replaced with the increase of others such as *Odoribacter*, the family of Christensenellaceae (phylum *Firmicutes*) and *Akkermansia* (in particular *muciniphila*), which are reduced in unhealthy aging (71). Gut colonization by *Akkermansia muciniphila* (*A. muciniphila*) may provide anti-inflammatory resilience and maintenance of gut barrier integrity, thus becoming a signature of healthy aging (72). *A. muciniphila* abundance can be found to be reduced in children with autism spectrum disorder (68, 73). According to some studies, it has emerged that the loss of GM diversity can be balanced by other commensals when individuals become “super-aged,” suggesting that the neuropathological processes underlying neurodegenerative diseases such as AD might be exacerbated when the age-dependent loss of GM diversity is not compensated by beneficial symbiotic bacteria.

An interesting connection between aging, GM senescence, and AD has emerged *via* investigations of the alterations of the amino acid L-tryptophan (Trp) and its metabolism. GM is essential in the production of different Trp metabolites. Trp is released from some dietary proteins, absorbed by the enterocytes, and made available in the GM as well as in immune and IECs where it exerts regulatory and protective functions against several inflammatory mechanisms (73). In the gut, Trp is responsible for the production of more than 90% of whole body serotonin (5-hydroxytriptamine, 5-HT) in the enterochromaffin cells *via* the rate-limiting enzyme Trp hydroxylase 1 enzyme (TPH1) (74, 75). Notably, some gut bacteria, and in particular *Clostridia*, can induce 5-HT production by promoting TPH1 gene expression (76). In addition, other commensals (e.g., *Streptococcus thermophilus* and *Lactobacillus plantarum*) are able to use Trp to produce 5-HT (77). To add complexity, 5-HT signaling can affect the immune response and act as an immunomodulator *via* the 5-HT receptors that have been identified in immune cells (78). The importance of these mechanisms has not only been recognized in different autoimmune disorders (78), but the higher susceptibility of the Aβ peptides released upon multiple challenges such as infection and depression has led to the re-conceptualization of AD itself as an autoimmune disease (79). In this view, Trp metabolites such as 5-HT are identified as effective bioactive molecules against the autoimmune processes triggered by Aβ oligomers (79).

Although peripheral 5-HT cannot cross the BBB, GM can affect central 5-HT synthesis by controlling the availability of Trp and also through different pathways, including microbial metabolites SCFAs (especially butyrate), Trp metabolites (e.g., tryptamine, indolic compounds, L-5-hydroxytryptophan), and by activating 5-HT receptors at terminals of vagal afferents communicating with brainstem neurons (80–82). The reduced 5-HT concentrations in the brains of AD patients (83) support the functional link between Trp metabolism in GM and the role of the brain-gut axis in AD pathogenesis. Decreased fecal Trp metabolites in the 5-HT pathway have been described in MCI and AD patients (84). Interestingly, antidepressants such as the selective 5-HT reuptake inhibitors (SSRIs) citalopram and escitalopram can reduce Aβ aggregates or Aβ plaque load in the cortex and/or hippocampus (85, 86). Remarkably, direct Trp supplementation *via* dietary intervention, increased 5-HT neurotransmission and 5-HT levels in the hippocampus and frontal cortex, preventing memory decline in aged animals (87). Similarly, high-Trp dietary supplementation was effective in reducing Aβ plaque in transgenic AD-like mice (88). A probiotic dietary supplementation, including *S. thermophilus*, some bifidobacteria (*B. longum, B. breve, B. infantis*), and selected lactobacilli (*L. acidophilus, L. plantarum, L. paracasei, L. delbrueckii subsp. Bulgaricus*, and *L. brevis*), were demonstrated to reduce Aβ aggregates and partially restore autophagy machinery in AD-like mice (89). Dietary supplementation with the commensal *Lactobacillus johnsonii* can reduce Trp catabolism and increase 5-HT concentrations in the intestinal ileum (90). In line with age-associated GM alterations and those described in AD patients and AD-like models, long-term dietary supplementation with a probiotic mix containing bifidobacteria (*B. lactis* and *B. bifidum*) and lactobacilli (*L. casei* and *L. acidophilus*) improved memory deficits and reduced age-associated disruption of BBB and intestinal barrier in a mouse model of accelerated senescence (91). Because of the increased conversion of Trp to kynurenine (Kyn) during immune activation and systemic inflammation, the increase of kynurenine/Trp ratio (KTR) is considered an index of enhanced Trp breakdown, which is increased with age (92) and higher than normal in AD patients (93), whose cognitive performance is inversely correlated to KTR upregulation (94). Along this line, there is to consider the regulatory role exerted by indoleamine 2,3-dioxygenase (IDO), which is the first rate-limiting enzyme initiating Trp catabolism along the Kyn pathway (95). IDO is highly expressed in immune cells and intestinal epithelium, and there is substantial evidence that alterations of Trp metabolism and excessive IDO activity are associated with chronic autoimmune diseases such as inflammatory bowel disease. Of note, there is evidence that the etiology of inflammatory bowel disease is associated with the pathogenetic interaction between the perturbation of GM diversity and the impact of IDO activity on the permeability of the intestinal barrier (96). IDO activity results were abnormally elevated in different chronic inflammatory states, including aging and obesity (97, 98), also producing immunosuppressive effects in different types of cancer and malignant conditions of the gastrointestinal tract (95, 99). A very well-designed study recently provided evidence not only of the association between obesity and an increase in intestinal IDO activity but also that genetic IDO deletion reduced endotoxemia and inflammation (98). In addition to 5-HT, GM bacteria can use Trp to produce indole derivatives or Trp catabolites (e.g., indole lactic acid) that are crucial for the preservation of mucosal barrier integrity *via* the activation of the aryl hydrocarbon receptor (AhR) (100). Acting as AhR ligands, these Trp-dependent metabolites are fundamental for GM homeostasis and provide higher resilience to intestinal inflammation, including inflammatory bowel disease (101). Notably, the AhR signaling induced by Trp metabolites has been shown to influence astrocyte activity and suppress brain inflammation (102). A decreased abundance of Trp-fermenting intestinal bacteria causing reduced production of Trp-derived indoles has been described in AD patients including, for instance, the decrease of indole-3-propionic acid (IPA)-producing *Bacteroides* and *Firmicutes* (103). Interestingly, IPA has shown neuroprotective and anti-oxidative potential against Aβ aggregation (104), and has been causally implicated in AhR upregulation and a parallel decrease of neuroinflammation in AD-like APP/PS1 mice (105).

Moreover, GM diversity can directly affect Trp metabolism and Trp serum levels, as well as alterations in Trp availability, which may have a drastic impact on bacterial abundance. There is a bidirectional interaction between Trp metabolism and the gut bacteria ecosystem, meaning changes in the composition of dietary macronutrients can reshape the abundance of selected bacteria. This is has been described for the consumption of the Mediterranean diet, which leads to an increase of *Lachnospiraceae* and Trp metabolites such as IPA (106). As a whole, it should also be noted that the control exerted by gut micro-organisms on Trp production and increase of Trp plasma levels (e.g., by *B. infantis*) (107) can directly influence 5-HT neurotransmission through the gut-brain axis. Indeed, the increase of Trp plasma levels after supplementation with *B. infantis* in rats is also accompanied by a decrease of 5-hydroxy indole acetic acid in the frontal cortex, which is an index of reduced 5-HT brain degradation.

The GM alterations in both the elderly and AD, together with the GM regulation of Trp metabolism and 5-HT synthesis, can allow us to view the comorbidity between AD-associated cognitive decline and NPSs from a “gut-centric” perspective. Moreover, the extraordinary capacity of the gut-brain axis in determining 5-HT homeostasis further contributes to linking the GM bionetwork to the emergence of NPSs in AD.

### Gut microbiota dysbiosis and serotonin neurotransmission dysfunctions: An essential link between Alzheimer’s disease and neuropsychiatric symptoms

**FIGURE 1:**
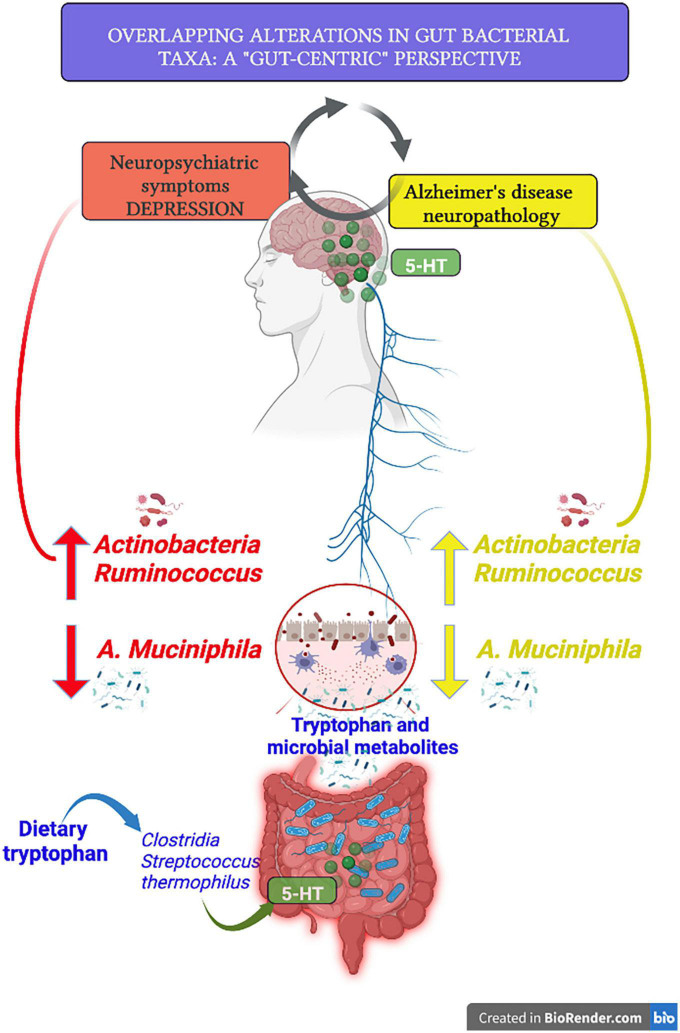
The figure depicts one of the issues reviewed, in particular some selective alterations found in the abundance of gut bacteria taxa in Alzheimer’s disease (AD) patients and in comorbidity with neuropsychiatric symptoms. Both depression and AD have been described as associated with a major increase of *Ruminococcus* and *Actinobacteria* and a parallel substantial reduction of gut colonization by *Akkermansia Muciniphila*. By the use of the dietary source of tryptophan, several gut microbiota (GM) members were recruited for the intestinal production of 5-hydroxytriptamine (5-HT), and multiple microbial (such as butyrate) and tryptophan (such as tryptamine and indolic compounds) metabolites contribute to the modulation of central 5-HT as well as to the activation of 5-HT at terminals of vagal afferents within the brainstem. The image was created by Biorender.com.

Apathy is the most recurrent NPSs in AD, followed by depression, anxiety, and sleep disturbances, while less frequently considered NPSs include irritability, hallucinations, and delusions (108). Depression is a risk factor that significantly increases the possibility of developing AD (109), including the association between LLD and the risk of dementia (110), and AD neuropathology. Thus, the clinical diagnosis and prognosis of dementia and AD are aggravated by depression (111). The use of SSRIs in AD patients is acknowledged to manage not only depressive symptoms but also contribute to preventing the exacerbation of cognitive decline, slowing down disease progression (112), and reducing conversion from MCI to AD (113). In AD-like mice models, SSRI treatment decreased both Aβ levels and Aβ load (85). Moreover, escitalopram administration reduced Aβ_42_ levels in the cerebrospinal fluid of healthy elderly subjects (114). The use of SSRIs is associated with a decreased rate of dementia/AD development (115). Notably, if the SSRI-based treatment is administered in non-cognitively impaired subjects who previously received a diagnosis of depression, the risk of AD development is either reduced or neutralized (116). In this view, the improvement of AD, the delay of cognitive aggravation, or the reduced risk of AD diagnosis might be (in part) attributed to the effects of SSRI treatment on GM. Indeed, there is robust evidence that antidepressants can shape GM and revert the disparity in gut bacterial diversity associated with depression. Upon improvement of depressive symptoms after escitalopram treatment, the composition of GM tends to resemble that of control subjects (117). The administration of different SSRIs in mice increased gut microbial diversity and reduced the abundance of *Ruminococcus*, while *Ruminococcus* (*flavefaciens*) dietary supplementation reinstated depressive-like behavior and abolished the effects of duloxetine treatment (118). Lately, a multi-omics study reported an elevated abundance of *Actinobacteria* and the abundance of *Ruminococcus* as discriminative bacteria of subjects with major depression (119). As for the increase of *Ruminococcus* and *Actinobacteria* species in depression, an increased abundance of *Actinobacteria* and *Ruminococcus* occurs in AD patients (Figure 1) (120).

As mentioned above, another way through which GM modulates the gut-brain axis is the production in the colon of transmitters (including 5-HT) and metabolites such as the SCFAs acetate, butyrate, and propionic acid. Upon its transport into blood circulation, butyrate exhibits neuroprotective potential contributing to restoring BBB integrity, reducing depression-like behaviors, and increasing brain 5-HT concentration (121). There might be a complex relationship between specific probiotics, SCFAs production, AD biomarkers, depression, and aging. *A. muciniphila* results increased in super-centenars (122) exerting a protective role of the gut barrier integrity (72), along with intestinal production of propionate and acetate (123). Recently, an improvement of spatial learning and a reduction of Aβ 40–42 levels in the cerebral cortex of APP/PS1 mice after *A. muciniphila* dietary intervention has been described (124), although a presymptomatic overexpression of *A. muciniphila* has also been reported in the same mouse model (125). *A. muciniphila* has been shown to have a beneficial impact on several inflammatory diseases, including obesity, metabolic syndrome, and type 2 diabetes (126). Considering inflammaging, AD, and depression, it is relevant that alterations of *A. muciniphila* abundance can be recurrent in these conditions (Figure 1) (127). The inverse relationship between *A. muciniphila* and depression is substantiated by animal models of adrenocorticotrophic hormone (ACTH)-induced, or social defeat-induced, depression- and anhedonic-like behaviors (128, 129). Analyzing the relationship between GM alterations and depression, nine bacteria genera were found to be increased in depressed patients (130). Among these, *Klebsiella*, *Streptococcus*, and *Lachnospiraceae* are progressively lost during aging.

The existence of cell-secreted particles and cell-released extracellular vesicles (EVs) (e.g., exosomes, microvesicles) acting as mediators for cell-to-cell communication has been recently extended to both gram-negative and gram-positive bacteria (131). These microbiota-secreted EVs (MEVs) can bypass (and regulate) the BBB, thus playing a pivotal role in the communication along the gut-brain axis (132) and, in particular, *A. muciniphila*-derived MEVs have been shown to protect against LPS-induced gut permeability (133) *A. muciniphila* probiotic supplementation and *A. muciniphila*-derived MEVs administration have been shown to increase 5-HT-mediated signaling in both the colon and hippocampus (134). The same authors demonstrated that the administration of *A. muciniphila* and *Faecalibacterium prausnitzii* (*F*. *prausnitzii*) and their derived MEVs, can increase both 5-HT levels and 5-HT transmission either potentiating the expression of genes involved in 5-HT synthesis in the enterochromaffin cells (e.g., *Tph1* gene) or inhibiting genes involved in 5-HT degradation (e.g., *MaoA* gene) (135).

Despite the robust link between GM homeostasis and 5-HT signaling, several mechanistic explanations underlying AD and depression comorbidity have been suggested in response to the importance of other signaling systems for AD pathogenesis, such as the excitatory glutamatergic system (136) and the remodeling of the inhibitory gamma-aminobutyric acid (GABA) circuits occurring during both the early and later stages of disease progression (137). Within this context, it is important to emphasize that alterations of GABA and glutamate signaling systems are associated with changes in selected GM members. For instance, a specific dietary intervention (i.e., ketogenic diet) was shown to increase the abundance of *A. muciniphila* and *Parabacteroides* and create a parallel increase in GABA/glutamate ratios in the hippocampus (138). Some bacterial strains can produce glutamate and convert it to D-glutamate, and these plasma levels are defective in AD patients (139).

Altogether, the dysfunction of Trp metabolism and 5-HT signaling driven by gut microbial dysbiosis and alteration of microbial diversity, or the significant changes in the abundance of selected bacteria, can help to account for the risk of AD neuropathology in the elderly, acting as a unifying concept underlying the incidence of comorbid cognitive decline and A-NPS (e.g., depression) in AD. The recognition of the importance that changes in 5-HT signaling and gut microbial ecosystem may have for the understanding of AD and depression comorbidity can also contribute to refining and potentiating diagnostic tools and the different options of intervention. Intriguingly, irritable bowel syndrome, enteropathies, and dysbiosis are frequent medical co-morbid conditions in patients with A-NPS and depression (140, 141). Hence, significant dietary modifications and personalized probiotic supplementation can be powerful tools of intervention in shaping GM composition. Awareness of this potential should be permanently introduced into clinical practice as a disease-modifying option during the initial stages of AD progression and symptoms of depression.

## Author contributions

RC conceived the study. RC and DC wrote the manuscript, which was revised by DD. DC, DD, and GG prepared the images. All authors critically discussed the manuscript.
